# Case report: Rapid symptom resolution of a mixed affective state with high-frequency repetitive transcranial magnetic stimulation

**DOI:** 10.3389/fpsyt.2023.1137055

**Published:** 2023-02-09

**Authors:** Emily M. Beydler, Lauren Katzell, Lauren Schmidt, Brent R. Carr, Richard C. Holbert

**Affiliations:** ^1^College of Medicine, University of Florida, Gainesville, FL, United States; ^2^Department of Psychiatry, University of Florida, Gainesville, FL, United States

**Keywords:** transcranial magnetic stimulation (TMS), bipolar disorder, mixed features specifier, bipolar (affective/mood) disorders, hypomania, major depression (MDD), treatment-refractory depression

## Abstract

**Introduction:**

Bipolar major depressive episodes with mixed features are diagnosed in patients who meet the full criteria for a major depressive episode exhibiting three additional concurrent symptoms of hypomania or mania. Up to half of patients with bipolar disorder experience mixed episodes, which are more likely to be treatment-refractory than pure depression or mania/hypomania alone.

**Case:**

We present a 68-year-old female with Bipolar Type II Disorder with a four-month medication-refractory major depressive episode with mixed features referred for neuromodulation consultation. Previous failed medication trials over several years included lithium, valproate, lamotrigine, topiramate, and quetiapine. She had no history of treatment with neuromodulation. At the initial consultation, her baseline Montgomery-Asberg Depression Rating Scale (MADRS) was moderate in severity at 32. Her Young Mania Rating Scale (YMRS) was 22, with dysphoric hypomanic symptoms consisting of heightened irritability, verbosity and increased rate of speech, and decreased sleep. She declined electroconvulsive therapy but elected to receive repetitive transcranial magnetic stimulation (rTMS).

**Interventions:**

The patient underwent repetitive transcranial magnetic stimulation (rTMS) with a Neuronetics NeuroStar system, receiving nine daily sessions over the left dorsolateral prefrontal cortex (DLPFC). Standard settings of 120% MT, 10 Hz (4 sec on, 26 sec off), and 3,000 pulses/session were used. Her acute symptoms showed a brisk response, and at the final treatment, her repeat MADRS was 2, and YMRS was 0. The patient reported feeling “great,” which she defined as feeling stable with minimal depression and hypomania for the first time in years.

**Conclusion:**

Mixed episodes present a treatment challenge given their limited treatment options and diminished responses. Previous research has shown decreased efficacy of lithium and antipsychotics in mixed episodes with dysphoric mood such as the episode our patient experienced. One open-label study of low-frequency right-sided rTMS showed promising results in patients with treatment-refractory depression with mixed features, but the role of rTMS in the management of these episodes is largely unexplored. Given the concern for potential manic mood switches, further investigation into the laterality, frequency, anatomical target, and efficacy of rTMS for bipolar major depressive episodes with mixed features is warranted.

## Introduction

Given the high prevalence, refractory nature, and mortality of mood episodes with mixed features in bipolar spectrum disorders, further research is needed to identify novel treatments. In particular, one promising modality is repetitive transcranial magnetic stimulation (rTMS), which has demonstrated efficacy for patients with treatment-refractory depression with mixed features. At the present, no randomized controlled trials have evaluated rTMS for bipolar mixed states, which merits careful study due to the risk of manic switches. This case of a patient who achieved full remission of an episode of bipolar depression with mixed features aims to explore possible therapeutic mechanisms, safety considerations, anatomical targeting, nosology, and future directions for mixed-state neuromodulation.

## Patient information

We present a 68-year-old female with Bipolar II Disorder suffering from a treatment-refractory episode of major depression (TRD) with mixed features referred for TMS consultation. She had been a high-functioning individual and was retired from a career in finance. She was originally diagnosed with Bipolar II Disorder at age 45 and has a history of multiple hypomanic episodes and a single psychiatric hospitalization for a major depressive episode. She has never had any suicide attempts. Despite the reduction of symptoms during her various acute episodes, her symptoms failed to ever achieve full remission for many years.

Previous treatment history included several years of medications that were either ineffective or merely transiently effective. Medication trials included lithium, valproate, lamotrigine, and quetiapine. Trials of adjunct antidepressants had occasional benefits. Unfortunately, her most beneficial treatment, lithium, was suspended following severe lithium-induced hypothyroidism that was treated with levothyroxine. She had no history of neuromodulation treatments of any modality. She declined the re-initiation of a mood stabilizer or antipsychotic given her frustration with previously unsuccessful trials. She also declined electroconvulsive therapy (ECT). Other medical history was significant for dyslipidemia, type II diabetes mellitus treated with combination sitagliptin-metformin, gastroesophageal reflux disease treated with omeprazole, and hypertension treated with furosemide and combination valsartan-hydrochlorothiazide.

The patient continued to decline ECT given concern for side effect profile, and as a result, the patient was ultimately referred for rTMS due to her unremitting, 4-month, mixed episode that was failing response despite a psychiatric medication regimen of clonazepam 1 mg TID, topiramate 50 mg BID, and gabapentin 200 mg TID.

## Clinical findings

Her depressive symptoms included depressed mood, anorexia, anhedonia, amotivation, poor concentration, and psychomotor agitation. She denied suicidal ideation. Hypomanic symptoms consisted of irritability, pressured speech, racing thoughts, distractibility, significant feelings of edginess and tension, and decreased need for sleep.

## Timeline


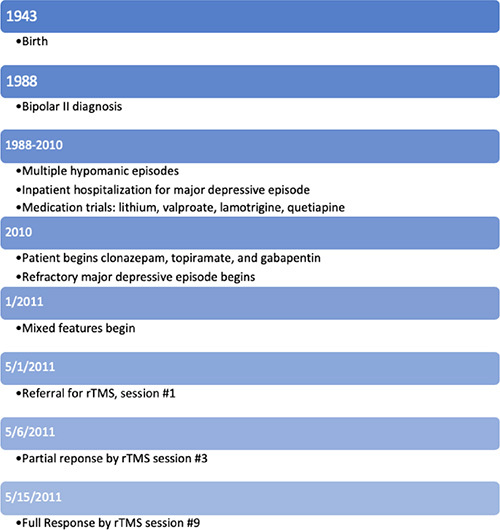


## Diagnostic assessment

At the time of presentation, her baseline Montgomery-Asberg Depression Rating Scale (MADRS) was 32 and Young Mania Rating Scale (YMRS) was 22. STMS was begun after obtaining informed consent, including a discussion of the possibility of inducing mania.

## Therapeutic intervention

Medications were held constant throughout her acute TMS treatment. She received nine daily sessions using the Neuronetics NeuroStar system. Treatment was administered over the left dorsolateral prefrontal cortex (DLPFC) with targeting fashioned after the standard Neurostar 5 cm rule (1). Similarly, the following standard settings were utilized: 120% MT, 10 Hz (4 sec ON, 26 sec OFF), and 3,000 pulses/session. She had a total of 9 treatment sessions on 9 separate days, with each TMS session consisting of 3,000 pulses, totaling 27,000 pulses over the course of treatment.

## Follow-up and outcomes

Our patient reported feeling better continuously throughout her TMS course, and irritability had subsided. A family member, who was around her most of the time, noted a significant benefit. The patient reported a noted response as early as TMS session #3; although encouraging, a placebo effect could not be ruled out. By TMS session #9 she reported feeling more stable than she had in years, with symptoms consistent with euthymia. Her reports of feeling stable were consistent with her MADRS of 2 and YMRS of 0. We offered a maintenance TMS taper, but the patient preferred to return home, to an area where there were no TMS psychiatrists. She followed up with her outpatient psychiatrist, and remained on the initial benzodiazepine dosage but discontinued all other pharmacotherapy. She experienced no symptoms of relapse in the year following her TMS course.

## Discussion

Bipolar disorder and its subtypes are chronic mood disorders affecting approximately 5% of the population (2, 3). Prominently classified in 1921 by Kraepelin (4) interest in bipolar disorder nosology traces back even further to the origins of psychiatric classification, with Hippocrates (460–337 BCE) identifying “melancholia, mania, and hypomania” (5). Kraepelin’s “mixed forms” of affect in bipolar disorder occur in 40% of patients with bipolar depression (3), and are at present classified in The Diagnostic and Statistical Manual of Mental Disorders, Fifth Edition (DSM V) as the specifier “with mixed features” (6). These episodes meet the full criteria for major depression, hypomania, or mania, with at least three symptoms of opposite polarity (6).

At baseline, patients with bipolar disorder have the highest rate of suicide of any psychiatric disorder, with rates 30 to 60 times higher than that of the general population at 20% (2). Mixed episodes further elevate suicide risk and are associated with higher rates of treatment resistance, comorbid medical and psychiatric illnesses, and decreased quality of life (2, 7, 8). There is no single pharmacologic agent indicated for mixed affective states of bipolar disorder based on randomized controlled trials (RCTs), with patients often trialing multiple medications with partial symptom improvement (3). The prevalence of polypharmacy in mixed states may result in decreased compliance and increased side effect profiles, such as lithium-induced hypothyroidism as in our patient. Management of lithium-induced hypothyroidism is consistent with that of primary hypothyroidism, with thyroxine initiation indicated for thyroid-stimulating hormone values > 10 mU/L (9). Given the straightforward treatment of lithium-induced hypothyroidism and the efficacy of lithium, its discontinuation is not recommended (9, 10). Patients like ours who decline lithium re-initiation should be presented with a thorough risk versus risk discussion, emphasizing the treatability of side effects and risks of uncontrolled mood episodes, before initiating an alternative mood stabilizer or another pharmacologic agent.

Despite their side effect profile, mood stabilizers including anticonvulsants remain first-line agents for mixed states, followed by atypical antipsychotics (3, 11). Thus, trialing a different mood stabilizer and then an atypical antipsychotic was recommended to our patient, but again she declined pharmacologic agents. Amongst mood stabilizers, lithium and lamotrigine may have decreased efficacy while valproate and carbamazepine have been shown to be effective in mixed states (3). While no RCTs have been performed using gabapentin and topiramate in mixed states, open-label studies have shown clinical benefit for patients, consistent with our patient’s partial improvement on these agents (3).

Antidepressant monotherapy is contraindicated due to the concern for manic switches, and chronic benzodiazepine usage is discouraged due to concerns for rebound anxiety, dependence, and agitation (3, 11). Our patient’s regimen of 3 mg of clonazepam daily at the time of presentation was not in line with these recommendations. Given her physiological dependence after a year on this medication, she declined dose adjustment at the time of the TMS consultation and we counseled her on tapering it with her outpatient psychiatrist. While benzodiazepines have been used to manage acute anxiety and agitation in refractory bipolar mania, recent work has shown that long-term use among bipolar benzodiazepine initiators is high, suggesting the need for caution in acute episodes given their concern for abuse potential and adverse side effects (12).

Electroconvulsive therapy, proven effective in both manic and depressive episodes of bipolar disorder, has also been reported to be highly effective in several refractory cases of mixed states (13). However, no standardized ECT protocol has been designed for mixed states, and no RCT has been conducted at this time (13, 14). Previous studies have shown equal response rates in bipolar and unipolar depression, low rates of manic switches, and up to 68% response to ECT in mixed states (13). Though ECT’s antidepressant and antimanic mechanisms remain unknown, the anticonvulsant hypothesis has been proposed as an explanation for ECT’s efficacy in bipolar disorder (15). Previous studies have shown decreased functional connectivity in the left DLPFC (Brodmann area 46) and adjacent Broca’s area (Brodmann areas 44 and 45) after ECT (16). This is consistent with the hyperconnectivity model of limbic dysregulation (16, 17). ECT has been posited to exert inhibitory effects as an anticonvulsant in frontal areas as opposed to its neurogenic effects seen in temporal areas, which in turn may also play a role in its mood stabilization properties (15).

Given concern for ECT’s side effect profile, wariness regarding the use of anesthesia, and the increasing availability of neuromodulation methods that do not elicit a seizure, patients like ours may elect to trial TMS off-label. However, it is prudent to have precautionary measures in place for off-label TMS, including the capability for inpatient hospitalization should symptoms worsen. Though side effects may be avoided with TMS, its response rates in unipolar depression remain inferior to those of ECT. TMS has been proven to be an effective treatment with minimal side effects for unipolar depression, for which it is FDA-approved (18, 19). Following Faraday’s Law, the TMS coil works by generating an alternating electric current, which discharges a magnetic field on the scalp resulting in an orthogonal electric field affecting cortical neurons to restore physiological rhythms that may be aberrant. High-frequency (10 Hz) TMS is thought to be excitatory, causing cortical neuron depolarization, and low-frequency (1 Hz) TMS is believed to be inhibitory, causing cortical neuron hyperpolarization (20). Given the durability of TMS after treatment, it is believed to exert effects through dopaminergic and glutamatergic neurotransmission, leading to lasting downstream long-term potentiation and depression (21).

Though robust literature exists on TMS for unipolar depression, at present, no RCTs have demonstrated efficacy of TMS for mania, hypomania, or mixed states (19, 22–24). However, various studies have shown promising related findings, utilizing TMS for treatment-resistant bipolar depression, maintenance treatment in bipolar disorder, and acute treatment in mixed states (25–28). One open-label study of 1 Hz right-DLPFC rTMS for mixed states showed promising preliminary findings (28). However, the risk of inducing mania with TMS remains equivocal and warrants further study (23, 29, 30). Choosing low-frequency stimulation to the right DLPFC target would have been reasonable for our patient. At the time this patient was treated, the literature on TMS in mixed states was even more scarce, so the decision was made to utilize the protocols in place for unipolar depression for technician consistency.

Though currently no clinical practice guidelines or validated protocols exist for TMS for bipolar depression, hypomania, mania, or mixed states, one meta-analysis showed that patients who underwent 10 Hz left-DLPFC rTMS had statistically significantly lower depression scores than 1 Hz right or bilateral DLPFC rTMS when compared to sham TMS (31). Another study found efficacy for bipolar depressive episodes using 10 Hz rTMS delivered to the left-DLPFC as well, with response and remission rates greater than those in unipolar depression (32). This is further corroborated by an observational study which found that 10 Hz rTMS delivered to the left-DLPFC in patients with bipolar depression had higher response rates versus patients with unipolar depression, especially for those on non-lithium mood stabilizers, such as our patient (33).

Previous work has shown that the predominant polarity across a patient’s lifetime, e.g., depression versus hypomania or mania, often guides clinician treatment selection in patients with bipolar spectrum disorders (34). Additionally, quantifying the predominant polarity in a “polarity index” has been shown to predict response to pharmacologic and psychotherapeutic treatments (34–36). However, this approach has not taken mixed features into account, with the majority of patients falling into the “undetermined predominant polarity” group, which suffers from higher aggression and relapse rates (34, 37, 38). The primary affective disturbance within a mixed episode may be a useful predictor of response to treatment (38). In line with this hypothesis, our patient’s dominant depression symptoms may have further contributed to her response to 10 Hz left-DLPFC rTMS, which is the approved protocol for unipolar major depression and has been demonstrated to be safe and effective for treatment-resistant unipolar depression (18).

Another possible factor in our patient’s response is the stimulation target. While initial estimates of DLPFC location based on the 5-cm target lack the precision and fidelity of more sophisticated Beam F3 or functional MRI (fMRI)-guided methods which yield a more anterolateral target, greater efficacy in bipolar disorder has resulted from using the 5-cm target (39, 40). Additionally, the 5-cm target has shown peak negative connectivity to the mania network map in the left DLPFC and peak positive connectivity in the right DLPFC (39).

Another theory that supports the role of predominant polarity in guiding treatment in bipolar spectrum disorders using neuromodulation is the frontal asymmetry hypothesis. Previous fMRI studies have shown asymmetrical cerebral hemisphere activation, with positive emotional valence associated with left hemisphere hyperactivity (decreases in prefrontal inhibitory alpha oscillations) and negative valence with right hemisphere hyperactivity in healthy controls (39, 41, 42). Additionally, lesion studies have shown right-hemispheric hypoactivity in mania and left-hemispheric hypoactivity in depression (39, 43–45). It follows that an approach to treating a patient with dominant manic symptoms would be exciting the hypofunctional area (right DLPFC) or inhibiting the hyperfunctional area (the left DLPFC). Previous studies have shown efficacy for 10 Hz right-DLPFC rTMS in mania (45), and additional work has shown negative connectivity between this stimulation target and the mania network map (39). At this time, no studies to our knowledge have examined hemispheric activation in mixed states. Given the concomitant nature of mixed episodes, further study of hemispheric asymmetry is needed to determine the role predominant symptom polarity plays in selecting a TMS treatment protocol for medication-refractory patients.

## Data availability statement

The original contributions presented in this study are included in this article/supplementary material, further inquiries can be directed to the corresponding author.

## Author contributions

BC and EB wrote the first draft of the manuscript and conceptualized the discussion section. RH provided the clinical resources for case report. All authors contributed to the manuscript editing, revision, read, and approved the submitted version.
